# Linearized and Kernelized Sparse Multitask Learning for Predicting Cognitive Outcomes in Alzheimer's Disease

**DOI:** 10.1155/2018/7429782

**Published:** 2018-01-24

**Authors:** Xiaoli Liu, Peng Cao, Jinzhu Yang, Dazhe Zhao

**Affiliations:** ^1^Computer Science and Engineering, Northeastern University, Shenyang, China; ^2^Key Laboratory of Medical Image Computing of Ministry of Education, Northeastern University, Shenyang, China

## Abstract

Alzheimer's disease (AD) has been not only the substantial financial burden to the health care system but also the emotional burden to patients and their families. Predicting cognitive performance of subjects from their magnetic resonance imaging (MRI) measures and identifying relevant imaging biomarkers are important research topics in the study of Alzheimer's disease. Recently, the multitask learning (MTL) methods with sparsity-inducing norm (e.g., *ℓ*_2,1_-norm) have been widely studied to select the discriminative feature subset from MRI features by incorporating inherent correlations among multiple clinical cognitive measures. However, these previous works formulate the prediction tasks as a linear regression problem. The major limitation is that they assumed a linear relationship between the MRI features and the cognitive outcomes. Some multikernel-based MTL methods have been proposed and shown better generalization ability due to the nonlinear advantage. We quantify the power of existing linear and nonlinear MTL methods by evaluating their performance on cognitive score prediction of Alzheimer's disease. Moreover, we extend the traditional *ℓ*_2,1_-norm to a more general *ℓ*_*q*_*ℓ*_1_-norm (*q* ≥ 1). Experiments on the Alzheimer's Disease Neuroimaging Initiative database showed that the nonlinear *ℓ*_2,1_*ℓ*_*q*_-MKMTL method not only achieved better prediction performance than the state-of-the-art competitive methods but also effectively fused the multimodality data.

## 1. Introduction

Alzheimer's disease (AD) is a severe neurodegenerative disorder that results in a loss of mental function due to the deterioration of brain tissue, leading directly to death [1]. It accounts for 60–70% of age related dementia, affecting an estimated 30 million individuals in 2011 and the number is projected to be over 114 million by 2050 [2]. The cause of AD is poorly understood and currently there is no cure for AD. AD has a long preclinical phase, lasting a decade or more. There is increasing research emphasis on detecting AD in the preclinical phase, before the onset of the irreversible neuron loss that characterizes the dementia phase of the disease, since therapies/treatment are most likely to be effective in this early phase. The Alzheimer's Disease Neuroimaging Initiative (ADNI, http://adni.loni.usc.edu/) has been facilitating the scientific evaluation of neuroimaging data including magnetic resonance imaging (MRI) and positron emission tomography (PET), along with other biomarkers and clinical and neuropsychological assessments for predicting the onset and progression of MCI (mild cognitive impairment) and AD. Early diagnosis of AD is key to the development, assessment, and monitoring of new treatments for AD.

Recently, rather than predicting categorical variables in the classification, various studies started to estimate continuous clinical variables from brain images. Therefore, instead of classifying a subject into binary or multiple predetermined categories or stages of the disease, regression focus is on estimating continuous values which may help to assess patient's disease progression. The most commonly used cognitive measures are Alzheimer's Disease Assessment Scale (ADAS) cognitive total score, Mini Mental State Exam (MMSE) score, and Rey Auditory Verbal Learning Test (RAVLT). Regression analyses were commonly used to predict cognitive scores from imaging measures. The relationship between commonly used cognitive measures and structural changes with MRI has been previously studied by regression models and the results demonstrated that there exists a relationship between baseline MRI features and cognitive measures [3, 4]. For example, Wan et al. proposed an elegant regression model called CORNLIN that employs a sparse Bayesian learning algorithm to predict multiple cognitive scores based on 98 structural MRI regions of interests (ROIs) for Alzheimer's disease patients. The polynomial model used in CORNLIN can detect either a nonlinear or a linear relationship between brain structure and cognitive decline [3]. Stonnington et al. adopted relevance vector regression, a sparse kernel method formulated in a Bayesian framework, to predict four sets of cognitive scores using MRI voxel based morphometry measures [4]. One of the biggest challenges in the prediction of inferring cognitive outcomes with MRI is the high dimensionality, which affects the computational performance and leads to a wrong estimation and identification of the relevant predictors. To reduce the high dimensionality and identify the relevant biomarkers, the sparse methods have attracted a great amount of research efforts in the neuroimaging field due to its sparsity-inducing property. Ye et al. applied sparse logistic regression with stability selection to ADNI data for robust feature selection [5] and successfully predicted the conversion from MCI into probable AD and identified a small subset of biosignatures.

It is known that there exist inherent correlations among multiple clinical cognitive variables of a subject. However, many works do not model dependence relation between multiple tasks and neglect the correlation between clinical tasks which is potentially useful. When the tasks are believed to be related, learning multiple related tasks jointly can improve the performance relative to learning each task separately. Multitask learning (MTL) is a statistical learning framework which aims at learning several models in a joint manner. It has been commonly used to obtain better generalization performance than learning each task individually [6, 7]. The critical issues in MTL are to identify how the tasks are related and build learning models to capture such task relatedness. The most recent studies [6, 8, 9] employed multitask learning with *ℓ*_2,1_-norm [7] regularization and aimed to select features that could predict all or most clinical scores. The *ℓ*_2,1_-norm is chosen to be the regularization. Thus, the *ℓ*_2,1_-norm regularized regression model is able to select some common features across all the tasks. However, in these learning methods, each task is traditionally performed by formulating a linear regression problem, in which the cognitive score is a linear function of the neuroimaging measures.

Kernel methods have been studied to model the cognitive scores as nonlinear functions of neuroimaging measures. Recently, many kernel-based classification or regression methods with faster optimization speed or stronger generalization performance have been proposed and investigated by theoretically analyzing and experimentally evaluating [10, 11]. Multiple kernel learning (MKL) [12], which learns the optimal kernel for a given task by a weighted, linear combination of predefined candidate kernels, has been introduced to handle the problem of kernel selection. The multiple kernel learning method not only learns an optimal combination of given base kernels but also provides a flexible framework to exploit the nonlinear relationship between MRI measures and cognitive scores.

In building the predictive model for classification or regression in AD, kernel has been widely used; therefore, it is important to extend the existing kernel-based learning methods to the case of multitask learning. In this paper, we propose two nonlinear multikernel-based multiple learning methods in [13] for building regression models, to exploit and investigate the nonlinear relationship between MRI measures and cognitive scores. Moreover, an *ℓ*_*q*_*ℓ*_1_-norm is used to extend the traditional *ℓ*_2_*ℓ*_1_-norm. The goal of our work is to (1) predict subjects' cognitive scores in a number of neuropsychological assessments using their MRI measures across the entire brain, (2) identify what the performance of the nonlinear method is compared with the linear *ℓ*_*q*_*ℓ*_1_-norm MTL and other MTL methods with different assumption. No previous studies have systematically and extensively examined the prediction performance by linear MTL and nonlinear MTL methods, and (3) identify what the learning capacity of the multikernel framework on fusing multimodality data is.

The rest of the paper is organized as follows. In Section 2, we provide a description of the multitask learning formulation. A linearized MTL and two multikernel-based MTL methods with *ℓ*_*q*_*ℓ*_1_-norm are provided in Section 3. In Section 4, we present the experimental results and compare the performance of linearized and kernelized MTL methods from the ADNI-1 dataset. The conclusion is drawn in Section 5.

## 2. Multitask Learning

Consider a multitask learning (MTL) setting with *T* tasks. Let *p* be the number of covariates, shared across all the tasks, and *m* be the number of samples. Let *X* ∈ *ℝ*^*m*×*p*^ denote the matrix of covariates, *Y* ∈ *ℝ*^*m*×*T*^ be the matrix of responses with each row corresponding to a sample, and Θ ∈ *ℝ*^*p*×*T*^ denote the parameter matrix, with column *θ*_.*t*_ ∈ *ℝ*^*p*^ corresponding to task *t*, *t* = 1,…, *T*, and row *θ*_*h*._ ∈ *ℝ*^*T*^ corresponding to feature *h*, *h* = 1,…, *p*.

The MTL formulation focuses on the following regularized loss function:

(1) minΘ∈Rp×T FY,X,Θ+λRΘ,

where *F*(·) denotes the loss function and *R*(·) is the regularizer. In the current context, we assume the loss to be square loss; that is,

(2) $$\begin{array}{c} F\left(\;Y,X,\Theta \;\right)={\left\|\;Y-X\Theta \;\right\|}_{F}^{2}=\overset{m}{\underset{i=1}{\sum }}{\left\|\;y_{i}-x_{i}\Theta \;\right\|}_{2}^{2}, \end{array}$$

where **y**_*i*_ ∈ *ℝ*^1×*T*^ and **x**_*i*_ ∈ *ℝ*^1×*p*^ are the *i*th rows of *Y* and *X*, respectively, corresponding to the multitask response and covariates for the *i*th sample. We note that the MTL framework can be easily extended to other loss functions. Base on some prior knowledge, we then add penalty *R*(Θ) to encode the relatedness among tasks.

## 3. *ℓ*_*q*_*ℓ*_1_-Norm Regularized Linearized Multitask Learning, *ℓ*_*q*_*ℓ*_1_-MTL

The *ℓ*_2_*ℓ*_1_-norm was popularly used in multitask feature learning [14]. All the existing algorithms for multitask feature learning assume a linear relationship between MRI features and cognitive scores and aim to learn a common subset of features for all tasks. Since the *ℓ*_2_*ℓ*_1_-norm regularizer imposes the sparsity between all features and nonsparsity between tasks, the features that are discriminative for all tasks will get large weights. However, the *ℓ*_2_*ℓ*_1_-norm is a fixed nonadaptive penalty. To obtain an adaptive regularization and better suit different data structures, we extend the *ℓ*_2,1_-norm to a larger class of mixed norm *ℓ*_*q*_*ℓ*_1_ that can be adapted to the data. The objective function of linear *ℓ*_*q*_*ℓ*_1_-MTL is formulated:

(3) minΘ 12Y−XΘF2+λΘq,1.

When *q* = 1, problem (3) reduces to the *ℓ*_1_-regularized problem; when *q* = 2, problem (3) reduces to the *ℓ*_2,1_-regularized problem.

An efficient algorithm is based on the accelerated gradient method for solving the *ℓ*_*q*_*ℓ*_1_-regularized problem, which is applicable for all values of *q* larger than 1.

First, construct the following model for approximating the composite function *ℳ*(·) at the point Θ^(*l*)^:

(4) ML,ΘlΘ≔FΘl+Θ−Θl,∇FΘl+L2Θ−ΘlF2+RΘ,

where *L* > 0. In the model *ℳ*_*L*,Θ^(*l*)^_(Θ), apply the first-order Taylor expansion at the point Θ (including all terms in the square bracket) for the smooth loss function *F*(·), and directly put the nonsmooth penalty *R*(·) into the model. The regularization term (*L*/2)‖Θ − Θ^(*l*)^‖_*F*_^2^ prevents Θ from walking far away from Θ^(*l*)^, and thus the model can be a good approximation to Φ(Θ) in the neighborhood of Θ^(*l*)^, where Φ(Θ) ≡ *F*(Θ) + *R*(Θ).

The accelerated gradient method is based on two sequences {Θ^(*l*)^} and {Γ^(*l*)^} in which {Θ^(*l*)^} is the sequence of approximate solutions and {Γ^(*l*)^} is the sequence of search points. The search point Γ^(*l*)^ is the affine combination of Θ^(*l*−1)^ and Θ^(*l*)^ as

(5) $$\begin{array}{c} \Gamma^{\left(l\right)}=\Theta^{\left(l\right)}+\beta^{\left(l\right)}\left(\;\Theta^{\left(l\right)}-\Theta^{\left(l-1\right)}\;\right), \end{array}$$

where *β*^(*l*)^ is a properly chosen coefficient. The approximate solution Θ^(*l*+1)^ is computed as the minimizer of *ℳ*_*L*^(*l*)^,Γ^(*l*)^_(Θ):

(6) Θl+1=arg minΘ MLl,ΓlΘ,

where *L*^(*l*)^ is determined by line search, for example, the Armijo-Goldstein rule, so that *L*^(*l*)^ should be appropriate for Γ^(*l*)^.

The key subroutine is (6), which can be computed as Θ^(*l*+1)^ = *π*_1*q*_(Γ^(*l*)^ − ∇*F*(Γ^(*l*)^)/*L*^(*l*)^, *λ*/*L*^(*l*)^), where *π*_1*q*_(·) is the *ℓ*_*q*_*ℓ*_1_-regularized Euclidean projection (EP_1*q*_) problem:

(7) π1qV,λ=argminΘ∈Rp×T 12Θ−VF2+λ∑h=1pθh.q.

Note that the *h* features in (7) are independent. In [15], the method can be used for ease of different independent groups; that is, *π*_1*q*_(*V*, *λ*) = arg min_*W*∈*ℝ*^*n*^_(1/2)‖*W* − *V*‖_2_^2^ + *λ*∑_*i*=1_^*𝒢*^‖*w*_*i*_‖_*q*_, where *𝒢* is the independent groups. In our paper, we focus on how the method deals with multitask learning problem in (7), where *𝒢* is equal to *p*, and each group denotes the corresponding feature shared across the multiple tasks. Thus, the optimization in (7) decouples into a set of *p* independent *ℓ*_*q*_-regularized Euclidean projection problems:

(8) πqvh.=argminθh.∈RT 12θh.−vh.22+λθh.q.

Then, the optimal solution *θ*_*h*._^*∗*^ of (8) can be gotten as follows:

(9) if vh.q¯≤λ, θh.∗=0;else  if vh.q¯≥λ,q=1, θh.∗=sgn⁡vh.⊙max⁡vh.−λ,0;else  if vh.q¯≥λ,q=2, θh.∗=vh.2−λvh.2vh.;else  if vh.q¯≥λ,q=∞, θh.∗=sgn⁡vh.⊙min⁡vh.,u∗;else vh.q¯≥λ,1<q<∞,  q≠2, θh.∗  is  the  unique  root  of  φc∗vh.,

where $\bar{q}=q/\left(q-1\right)$, and thus *q* and $\bar{q}$ satisfy the following relationship: $1/\bar{q}+1/q=1$, *u*^*∗*^ is the unique root of *ζ*(*u*) = ∑_*h*=1_^*p*^max⁡(|*v*_*h*._| − *u*, 0) − *λ*, and *ζ*(·) is an auxiliary function, defined as *ζ*_*c*_^*v*^(*θ*) = *θ* + *cθ*^*q*−1^ − *v* with 0 ≤ *θ* ≤ *v*; And *φ*_*c*_^*v*^(*θ*) = *θ* + *cθ*^(*q*−1)^ − *v*, 0 < *x* < *v* and *c*^*∗*^ = *λ*‖*θ*_*h*._^*∗*^‖_*q*_^1−*q*^. Note that **z** = **x**⊙**y** denotes *z*_*i*_ = *x*_*i*_*y*_*i*_.

The algorithm *ℓ*_*q*_*ℓ*_1_-MTL is summarized in Algorithm 1.

## 4. Kernelized Multitask Learning

### 4.1. Multikernel Learning

The limitation in this traditional *ℓ*_2,1_-norm MTL model is that subjects cognitive score under a task is modeled as a linear function of his/her MRI measures. The kernel methods, for example, SVM or SVR, can model the nonlinear distribution of the data by mapping the input data into a nonlinear feature space by kernel embedding. In this section, we consider the case that *ℓ*_2,1_-norm regularized MTL is extended to kernel method. Let us define the kernel function $\phi_{j}(x):{\mathbb{R}}^{p}\to {\mathbb{R}}^{\hat{p}}$, which maps the data samples from an input space to a feature space (a high-dimensional Hilbert space *ℋ*), where $\hat{p}$ denotes the dimensionality of the feature space and **x** is a sample from the input space. A kernel function *k* is capable of attaining the inner product of two mapped datasets in *ℋ*: *k*(**x**, **x**′) = *ϕ*(**x**) · *ϕ*(**x**′) in the original space without explicitly computing the mapped data. The associated Gram matrix has entries *K*(*i*, *j*) = *k*(**x**, **x**′).

The most suitable types and parameters of the kernels for a particular task are often unknown, and the selection of the optimal kernel by exhaustive search on a predefined pool of kernels is usually time-consuming and sometimes causes overfitting. Multiple kernel learning (MKL) attempts to achieve better results by combining several base kernels instead of using only one specific kernel. MKL assumes that **x**_*i*_ can be mapped to *k* different Hilbert spaces, **x**_*i*_ → *ϕ*_*j*_(**x**_*i*_), *j* = 1,…, *k*, implicitly with *k* nonlinear mapping functions, and the objective of MKL is to seek the optimal kernel combination $\hat{k}(x,x^{\prime })=\sum_{j=1}^{k}d_{j}k_{j}(x,x^{\prime }),\;\;d_{j}\geq 0,\;\;\sum_{j=1}^{k}d_{j}=1$, where **d** is the kernel weight vector. The primal objective function of multiple kernel regression model is written as follows:

(10) minθ~,ξ 12∑j=1kθ~j22dj+λ2∑i=1mξi2,s.t. ∑j=1kθ~jTϕjxi−yi=ξi, ∑j=1kdj=1,dj≥0.

MKL learns both the weights of the kernel combination **d** and the parameters of the regression $\tilde{\theta }$ by solving a single joint optimization problem.

Using **α** to denote the Lagrange multipliers, the objective value of the dual problem of (10) can be written as follows:

(11) Jd=maxα −αTyt−12αTK^α−12Cα∗Tα,s.t. ∑j=1kdj=1,dj≥0,

where $\hat{K}=\sum_{j=1}^{k}d_{j}K_{j}$ is the combined Gram matrix and *K*_*j*_, *j* = 1,…, *k*, is the given set of base kernels.

### 4.2. *ℓ*_*q*_*ℓ*_1_-Norm Regularized Multikernel Multitask Learning, *ℓ*_*q*_*ℓ*_1_-MKMTL

We follow the multiple kernel learning scheme and use the *ℓ*_*q*,1_-norm to model the relationship between the tasks to learn a common kernel representation by imposing sparsity constraint on the kernel weight. The method, called *ℓ*_*q*_*ℓ*_1_-MKMTL, assumes that few base kernels are important for the tasks and encourages a linear combination of only few kernels and assumes few selected kernels are similar across the tasks. The formulation of *ℓ*_*q*_*ℓ*_1_-MKMTL can be expressed as follows:

(12) minθ~,ξ 12∑j=1k∑t=1Tθ~tj2q1/q2+λ2∑t=1T∑i=1mtξti2,s.t. ∑j=1kθ~tjTϕjxti−yti=ξti.

We now rewrite this formulation in a convenient form which can be efficiently solved using mirror-descent based algorithms. We introduce some more notations: let Δ_*d*,*r*_ = {**z** ≡ [*z*_1_,…, *z*_*d*_]^*T*^∣∑_*i*=1_^*d*^*z*_*i*_^*r*^ ≤ 1, *z*_*i*_ ≥ 0, *i* = 1,…, *d*} and with slight abuse of notation let Δ_*d*,1_ = Δ_*d*·_. Next, we note the following [16].

**Lemma 1 .**

Let *a*_*i*_ ≥ 0, *i* = 1,…, *d* and 1 < *r* < *∞*. Then, for Δ_*d*,*r*_ defined as before,

(13) minη∈Δd,r ∑iaiηi=∑i=1dair/r+1r+1/r,

and the minimum is attained at

(14) $$\begin{array}{c} \eta_{i}=\frac{a_{i}^{1/\left(r+1\right)}}{{\left(\sum_{i=1}^{d}a_{i}^{r/\left(r+1\right)}\right)}^{1/r}}, \end{array}$$

with the convention that *a*/0 is 0 if *a* = 0 and is *∞* if *a* ≠ 0.

Using the result of the lemma (with *r* = 1) and introducing variables *μ* = [*μ*_1_,…, *μ*_*k*_]^*T*^, we have

(15) ∑j=1k∑t=1Tθ~tj2q1/q2=minμ∈Δk⁡∑j=1k∑t=1Tθ~tj2q2/qμj.

Now introducing dual variables *ν*_*j*_ = [*ν*_*j*1_,…, *ν*_*jT*_]^*T*^, *j* = 1,…, *k*, and using the notion of dual norm [17], we obtain

(16) $$\begin{array}{c} {\left(\;\sum_{t=1}^{T}{\left(\;{\left\|\;{\tilde{\theta }}_{tj}\;\right\|}_{2}^{2}\;\right)}^{q/2}\;\right)}^{2/q}=\underset{\nu_{j}\in \Delta_{T,\bar{q}}}{\max}\;\overset{T}{\underset{t=1}{\sum }}\nu_{jt}{\left\|\;{\tilde{\theta }}_{tj}\;\right\|}_{2}^{2}, \end{array}$$

where $\bar{q}=q/\left(q-2\right)$. With this, the objective in the *ℓ*_*q*_*ℓ*_1_-MKMTL formulation can now be written as

(17) minμ∈Δk minθ~,ξ maxνj∈ΔT,q¯ 12∑j=1k∑t=1Tνjtθ~tj22μj+λ2∑t=1T ∑i=1mtξti2.

Using *α* to denote the Lagrange multipliers, this has the Lagrangian

(18) L=12∑j=1k∑t=1Tνjtθ~tj22μj+λ2∑t=1T ∑i=1mtξti2+∑t=1T ∑i=1mtαti∑j=1kθ~tjTϕjxti−yti−ξti.

Recall our foray into Lagrange duality. We can solve the original problem by doing

(19) maxα minθ~,ξ Lθ~,ξ,α.

To begin, we attack the inner minimization: For fixed *α*, we would like to solve for the minimizing $\tilde{\theta }$ and *ξ*. We can do this by setting the derivatives of *ℒ* with respect to *ξ*_*ti*_ and $\tilde{\theta }$ to be zero. Doing this, we can find

(20a) θ~tj∗=−αtT∑j=1kμjΦtjνjt,

(20b) ξti∗=αtiλ,

where *α*_*t*_ is a vector corresponding to the *t*th task in the *ℓ*_*q*_*ℓ*_1_-MKMTL formulation and Φ_*tj*_ is the data matrix with columns as *ϕ*_*j*_(*x*_*ti*_), *i* = 1,…, *m*_*t*_. So, we can solve the problem by maximizing the Lagrangian (with respect to *α*), where we substitute the above expressions for *ξ* and $\tilde{\theta }$. Thus, we have an unconstrained maximization.

(21) maxα ∑t=1T−αtTyt−12αtT∑j=1kμjKtjνjtαt−12λαtTαt.

Here, **y**_*t*_ is vector of scores of the *t*th task training data points and **K**_*ij*_ represents the Gram matrix of the *t*th task training data points with respect to the *j*th kernel. Equation (21) is just a quadratic in *α*. As such, we can find the optimum as the solution of a linear system.

Then, (17) can be written as follows:

(22) minμ∈Δk maxνj∈ΔT,q¯ maxα ∑t=1T−αtTyt−12αtT∑j=1kμjKtjνjtαt−12λαtTαt.

The formulation can be transformed as follows:

(23) minμ∈Δk maxνj∈ΔT,q¯ maxα ∑t=1T−αtTyt−12αtT∑j=1kμjKtjνjtαt−12λαtTαt.

The algorithm *ℓ*_*q*_*ℓ*_1_-MKMTL is summarized in Algorithm 2.

### 4.3. *ℓ*_2,1_-*ℓ*_*q*_-Norm Regularized Multikernel Multitask Learning, *ℓ*_2,1_*ℓ*_*q*_-MKMTL

The linearized *ℓ*_*q*_*ℓ*_1_-MTL assumed linear relationship between the MRI features and the cognitive outcomes. Such a model is the lack of capability to capture nonlinear predictive information from the features. Although the *ℓ*_*q*_*ℓ*_1_-MKMTL builds the nonlinear relationship for the features and task by mapping to high-dimensional space, it considers that tasks to be learned share a common subset of kernel representations without capturing the interrelationships between different cognitive measures over the feature space.

To overcome the weaknesses of the previous two methods, we project the original feature vectors to a high-dimensional space using multiple nonlinear mapping functions for performing regression task in a nonlinear manner and utilize multitask learning in the multiple kernel spaces for modeling the disease's cognitive scores with a joint *ℓ*_2,1_-*ℓ*_*q*_ sparsity-inducing regularizers. Moreover, we construct new features as orthogonal transforms of the given features, that is, **L**_*j*_*ϕ*_*j*_(*x*), where **L**_*j*_ is an orthogonal matrix which is to be learned. Again, low empirical risk over each task would imply minimizing the following quadratic loss: $\sum_{t=1}^{T}\sum_{i=1}^{m_{t}}\min{\left(\sum_{j=1}^{k}{\tilde{\theta }}_{tj}^{T}L_{j}^{T}\phi_{j}\left(x_{ti}\right)-y_{ti}\right)}^{2}$. Before describing the regularization term, we introduce some more notations: Let the entries of ${\tilde{\theta }}_{tj}$ be ${\tilde{\theta }}_{tjl},\;\;l=1,\ldots ,p_{j}$, where *p*_*j*_ is the dimensionality of the feature space induced by the *j*th kernel. By ${\tilde{\theta }}_{.jl}$ we denote the vector with entries ${\tilde{\theta }}_{tjl},\;\;t=1,\ldots ,T$. The regularization term we employ is ${\left(\sum_{j=1}^{k}{\left(\sum_{l=1}^{p_{j}}{\left\|{\tilde{\theta }}_{.jl}\right\|}_{2}\right)}^{q}\right)}^{2/q}$, where *q* ∈ [1,2]. Different from *ℓ*_*q*_*ℓ*_1_-MKMTL, the *ℓ*_*q*_-norm in *ℓ*_2,1_*ℓ*_*q*_-MKMTL is employed over the kernels rather than the tasks.

Mathematically, the *ℓ*_2,1_*ℓ*_*q*_-MKMTL formulation can be expressed as follows:

(24) minθ~,ξ,L 12∑j=1k∑l=1pjθ~.jl2q2/q+λ2∑t=1T∑i=1mtξti2,s.t. ∑j=1kθ~tjTLjTϕjxti−yti=ξti,Lj∈Opj,

where *O*^*p*_*j*_^ represents the set of all orthogonal matrices of dimensionality *p*_*j*_. In the following text, we rewrite this formulation in a form which is convenient to solve using an MD based algorithm.

Using the result of Lemma 1 and introducing new variables *ν* = [*ν*_1_,…, *ν*_*k*_]^*T*^, we have

(25) $$\begin{array}{c} {\left(\;\sum_{j=1}^{k}{\left(\;\sum_{l=1}^{p_{j}}{\left\|\;{\tilde{\theta }}_{.jl}\;\right\|}_{2}\;\right)}^{q}\;\right)}^{2/q}=\underset{\nu \in \Delta_{k,\bar{q}}}{\min}\;\overset{k}{\underset{j=1}{\sum }}\frac{{\left(\sum_{l=1}^{p_{j}}{\left\|{\tilde{\theta }}_{.jl}\right\|}_{2}\right)}^{2}}{\nu_{j}}, \end{array}$$

where $\bar{q}=q/\left(2-q\right)$. Again using the lemma and introducing new variables *μ*_*j*_ = [*μ*_*j*1_,…, *μ*_*jp*_*j*__]^*T*^, *j* = 1,…, *k*, the regularizer can be written as

(26) minν∈Δk,q¯ minμj∈Δpj ∑t=1T∑j=1k∑l=1pjθ~tjl2μjkνj.

Now, we perform a change of variables: ${\tilde{\theta }}_{tjl}/\sqrt{\mu_{jk}\nu_{j}}={\bar{\theta }}_{tjl},\;\;l=1,\ldots ,p_{j}$. Using this, one can rewrite the *ℓ*_2,1_*ℓ*_*q*_-MKMTL formulation as

(27) minν,μj,Lj ∑t=1Tminθ¯t,ξt⁡12∑j=1kθ¯tjTθ¯tj+λ2∑t=1T∑i=1mtξti2,s.t. ∑j=1kθ¯tjTΛj1/2LjTϕjxti−yti=ξti, ν∈Δk,q¯,  μj∈Δpj,  Lj∈Opj,

where Λ_*j*_ is a diagonal matrix with entries as *ν*_*j*_*μ*_*jl*_, *l* = 1,…, *p*_*j*_.

Now, using *α* to denote the Lagrange multipliers, this has the Lagrangian of

(28) L=∑t=1T12∑j=1kθ¯tjTθ¯tj+λ2∑i=1mtξti2+∑i=1mtαti∑j=1kθ¯tjTΛj1/2LjTϕjxti−yti−ξti.

This can be solved like *ℓ*_*q*_*ℓ*_1_-MKMTL:

(29a) θ~tj∗=−αtTΛj1/2LjTΦtj,

(29b) ξti∗=αtiλ.

Again, we substitute the above expressions for *ξ* and $\tilde{\theta }$. Thus, we have the following form:

(30) minν,μj,Lj ∑t=1Tmaxαt⁡−αtTyt−12αtT∑j=1kΦtjTLjTΛjLjΦtjαt−12λαtTαts.t. ν∈Δk,q¯,  μj∈Δpj,  Lj∈Opj.

Denoting **L**_*j*_^*T*^Λ_*j*_**L**_*j*_ by ${\bar{Q}}_{j}$ and eliminating variables *ν*, *μ*, and **L**'s lead to

(31) minQ¯ ∑t=1Tmaxαt⁡−αtTyt−12αtT∑j=1kΦtjTQ¯jΦtjαt−12λαtTαts.t. Q¯j⪰0,  ∑j=1ktrQ¯jq¯≤1.

The difficulty in working with this formulation is that the explicit mappings *ϕ*_*j*_'s are required. We now describe a way of overcoming this problem and efficiently kernelizing the formulation (refer to [1] also). Let Φ_*j*_ ≡ [Φ_1*j*_,…, Φ_*Tj*_] and the compact SVD of Φ_*j*_ be **U**_*j*_Σ_*j*_**V**_*j*_^*T*^. Then, we introduce a symmetric positive semidefinite **Q**_*j*_ with the same rank as that of Φ_*j*_ such that ${\bar{Q}}_{j}=U_{j}Q_{j}U_{j}^{T}$. By eliminating ${\bar{Q}}_{j}$, we can rewrite the above problem using **Q**_*j*_ as

(32) minQ ∑t=1Tmaxαt⁡−αtTyt−12αtT∑j=1kMtjTQjMtjαt−12λαtTαts.t. Qj⪰0,  ∑j=1ktrQjq¯≤1,

where **M**_*tj*_ = Σ_*j*_^−1^**V**_*j*_^*T*^Φ_*j*_^*T*^Φ_*tj*_. Note that calculation of **M**_*tj*_ does not require the kernel-induced features explicitly and hence the formulation is kernelized. It can be transformed as follows:

(33) minQ fQ=∑t=1T−αtTyt−12trQB−12λαtTαt,

where **B** is a block diagonal matrix with entries as **B**_*j*_ = ∑_*t*=1_^*T*^**M**_*tj*_*α*_*t*_*α*_*t*_^*T*^**M**_*tj*_^*T*^.

**Q** can be solved by mirror-descent. The gradient of ∇*f* with respect to **Q** is calculated as follows:

(34) $$\begin{array}{c} \nabla f\left(\;Q^{\left(l\right)}\;\right)=-\frac{1}{2}B^{\left(l\right)}, \end{array}$$

where **B**^(*l*)^ is the value obtained using optimal *α*_*t*_ obtained while evaluating *f*(**Q**^(*l*)^).

The algorithm *ℓ*_2,1_-*ℓ*_*q*_ MKMTL is summarized in Algorithm 3.

## 5. Experimental Results and Discussions

### 5.1. Experimental Setup

We use 10-fold cross valuation to evaluate our model and conduct the comparison. In each of ten trials, a 5-fold nested cross validation procedure is employed to tune the regularization parameters. Data was *z*-scored before applying regression methods. The range of each parameter varied from 10^−1^ to 10^3^. The candidate kernels are as follows: six different kernel bandwidths (2^−2^, 2^−1^,…, 2^3^), polynomial kernels of degrees 1 to 3, and a linear kernel, which totally yields 10 kernels. The kernel matrices were precomputed and normalized to have unit trace. The reported results were the best results of each method with the optimal parameter. For the quantitative performance evaluation, we employed the metrics of Correlation Coefficient (CC) and Root Mean Squared Error (rMSE) between the predicted clinical scores and the target clinical scores for each regression task. Moreover, to evaluate the overall performance on all the tasks, the normalized mean squared error (nMSE) [7, 18] and weighted R-value (wR) [4] are used. The nMSE and wR are defined as follows:

(35) nMSEY,Y^=∑t=1TYt−Y^t22/σYt∑t=1Tmt,

(36) wRY,Y^=∑t=1TCorrYt,Y^tmt∑t=1Tmt,

where *Y* and $\hat{Y}$ are the ground truth cognitive scores and the predicted cognitive scores, respectively.

A smaller (higher) value of nMSE and rMSE (CC and wR) represents better regression performance. We report the mean and standard deviation based on 10 iterations of experiments on different splits of data for all comparable experiments.

In ADNI, all participants received 1.5-Tesla (T) structural MRI. The MRI features used in our experiments are based on the imaging data from the ADNI database processed by a team from UCSF (University of California at San Francisco), who performed cortical reconstruction and volumetric segmentations with the FreeSurfer image analysis suite (http://surfer.nmr.mgh.harvard.edu/) according to the atlas generated in [19]. Totally, 48 cortical regions and 44 subcortical regions are generated. For each cortical region, the cortical thickness average (TA), standard deviation of thickness (TS), surface area (SA), and cortical volume (CV) were calculated as features. For each subcortical region, subcortical volume was calculated as features. The SA of left and right hemisphere and total intracranial volume (ICV) were also included. This yielded a total of *p* = 319 MRI features extracted from cortical/subcortical ROIs in each hemisphere (including 275 cortical and 44 subcortical features). Details of the analysis procedure are available at http://adni.loni.usc.edu/methods/mri-analysis/.

Ten widely used clinical/cognitive assessment scores [3, 20, 21] were employed in this study, including Alzheimer's Disease Assessment Scale (ADAS) cognitive total score, Mini Mental State Exam (MMSE) score, Rey Auditory Verbal Learning Test (RAVLT) involving total score of the first 5 learning trials (TOTAL), Trial 6 total number of words recalled (TOT6), 30-minute delay score (T30), and 30-minute delay recognition score (RECOG), FLU involving animal total score (ANIM) and vegetable total score (VEG), and TRAILS including Trail Making test A score and B score.

### 5.2. Comparison with the State-of-the-Art MTL Methods

To compare the kernelized MTL with the other linearized one and illustrate how well the two multikernel-based MTL methods work by means of modeling the correlation among the tasks, we comprehensively compare our proposed methods with several popular state-of-the-art related methods. Representative comparable algorithms include

1. Ridge [22]: min_Θ_ *L*(*X*, *Y*, Θ) + *λ*‖Θ‖_*F*_^2^
2. Lasso [23]: min_Θ_ *L*(*X*, *Y*, Θ) + *λ*‖Θ‖_1_
3. MKL [24]: ${min}_{\tilde{\theta },\xi }\;\left(1/2\right){\left\|f\right\|}_{ℋ}^{2}+\lambda \sum_{i}\xi_{i}$, such that *y*_*i*_(*f*(*x*_*i*_) + *b*) ≥ 1 − *ξ*_*i*_ and *ξ*_*i*_ ≥ 0, ∀*i*
4. Robust Multitask Feature Learning (RMTL) [25]: RMTL (min_Θ_ *L*(*X*, *Y*, Θ) + *λ*_1_‖*P*‖_*∗*_ + *λ*_2_‖*S*‖_2,1_, subject to Θ = *P* + *S*), which assumes that the model Θ can be decomposed into two components: a shared feature structure *P* capturing task relatedness and a group-sparse structure *S* detecting outliers
5. Clustered Multitask Learning (CMTL) [16]: CMTL (min_Θ,*M*:*M*^*T*^*M*=*I*_*c*_ _*L*(*X*, *Y*, Θ) + *λ*_1_(tr(Θ^*T*^Θ) − tr(*M*^*T*^Θ^*T*^Θ*M*)) + *λ*_2_tr(Θ^*T*^Θ), where *M* ∈ *ℝ*^*c*×*k*^ is an orthogonal cluster indicator matrix and the tasks are clustered into *c* < *k* clusters) incorporating a regularization term to induce clustering between tasks and then sharing information only to tasks belonging to the same cluster. In the CMTL, the number of clusters is set to 11 since the 20 tasks belong to 11 sets of cognitive functions
6. Trace-norm regularized multitask learning (Trace) [17]: assuming that all models share a common low-dimensional subspace (min_Θ_ *L*(*X*, *Y*, Θ) + *λ*‖Θ‖_*∗*_)
7. Sparse regularized multitask learning formulation (SRMTL) [26]: SRMTL (min_Θ_ *L*(*X*, *Y*, Θ) + *λ*_1_‖Θ*𝒵*‖_*F*_^2^ + *λ*_2_‖Θ‖_1_, where *𝒵* ∈ *ℝ*^*T*×*T*^) containing two regularization processes: (1) all tasks are regularized by their mean value, and therefore knowledge from one task can be utilized by other tasks via the mean value; (2) sparsity is enforced in the learning with *ℓ*_1_-norm.

Experimental results are reported in Tables 1 and 2 where the best results are boldfaced. A first glance at the results shows that *ℓ*_2,1_*ℓ*_*q*_-MKMTL generally outperforms all the other compared methods on both metrics and across all the cognitive tasks. Additionally, a statistical analysis is performed on the results. As can be seen, our proposed method achieves statistically significant results compared to all the other methods on most of the results. These results reveal several interesting points:

1. All the compared multitask learning methods (*ℓ*_*q*_*ℓ*_1_-MTL, *ℓ*_*q*_*ℓ*_1_-MKMTL, and *ℓ*_2,1_*ℓ*_*q*_-MKMTL) improve the predictive performance over the independent regression algorithms (Ridge, Lasso, and MKL). This justifies the motivation of learning multiple tasks simultaneously.
2. The two multikernel-based MTL methods outperform the linearized *ℓ*_*q*_*ℓ*_1_-MTL in terms of nMSE, and *ℓ*_2,1_*ℓ*_*q*_-MKMTL outperforms the linearized *ℓ*_*q*_*ℓ*_1_-MTL in terms of wR. It indicates that the nonlinear MTL models via kernel functions can capture complex patterns between brain images and the corresponding cognitive measures.
3. By the appropriate *ℓ*_2,1_*ℓ*_*q*_ regularization, the *ℓ*_2,1_*ℓ*_*q*_-MKMTL model enables us (1) to obtain capture nonlinear associations between MRI and cognitive outcomes, (2) to obtain the intrinsic relationships between multiple related tasks in *ℋ*, and (3) to promote the sparse kernel combinations to support the interpretability and scalability. The outcomes demonstrate that *ℓ*_2,1_*ℓ*_*q*_-MKMTL outperforms *ℓ*_*q*_*ℓ*_1_-MTL and *ℓ*_*q*_*ℓ*_1_-MKMTL, both of which neglect the inherently nonlinear relationship between MRI and cognitive outcomes, and the correlation among multiple related tasks in the feature space.
4. Compared with the other multitask learning methods with different assumptions, our proposed methods belong to the multitask feature learning methods with sparsity-inducing norms, having an advantage over the other comparative multitask learning methods. Since not all the brain regions are associated with AD, many of the features are irrelevant and redundant. Sparse based MTL methods are appropriate for the task of predicting cognitive measures and better than the non-sparse-based MTL methods.

**Figure 1:**
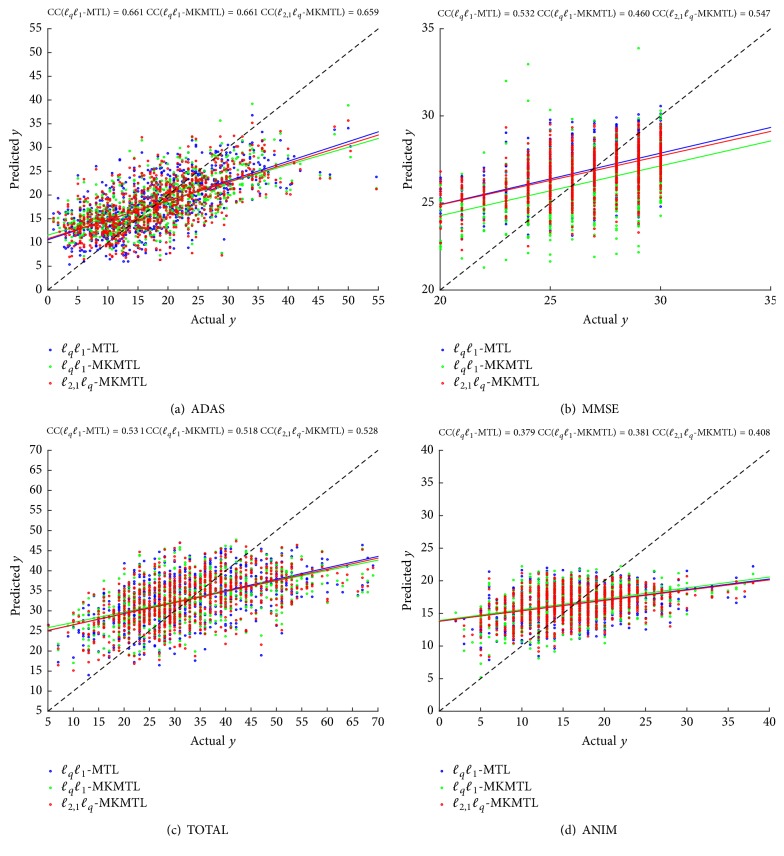
Scatter plots of actual versus predicted values of cognitive scores on each fold testing data using three comparable MTL methods based on MRI features.

We also show the scatter plots of actual values versus predicted values for the score of ADAS, MMSE, TOTAL, and ANIM on testing data in Figure 1.

### 5.3. Multimodalities Fusion

To estimate the effect of combining multimodality image data with the linearized and kernelized MTL methods and provide a more comprehensive comparison of the results from the comparable MTL models, we further perform some experiments, and they are (1) using only MRI modality, (2) using only PET modality, (3) combining two modalities: PET and MRI (MP), and (4) combining three modalities: PET, MRI, and demographic information including age, gender, years of education, and ApoE genotyping (MPD). Different from the above experiments, the samples from ADNI-2 are used instead of ADNI-1, since the amount of the patients with PET is sufficient. From the ADNI-2, we obtained all the patients with both MRI and PET, totally 756 samples. The PET imaging data are from the ADNI database processed by the UC Berkeley team, who use a native-space MRI scan for each subject that is segmented and parcellated with FreeSurfer to generate a summary cortical and subcortical ROI, and they coregister each florbetapir scan to the corresponding MRI and calculate the mean florbetapir uptake within the cortical and reference regions. The procedure of image processing is described in http://adni.loni.usc.edu/updated-florbetapir-av-45-pet-analysis-results/. In the *ℓ*_*q*_*ℓ*_1_-MKMTL and *ℓ*_2,1_*ℓ*_*q*_-MKMTL, ten different kennel functions described in the first experiment are used for each modality. To show the advantage of the kernel-based methods, we compare them with linear *ℓ*_*q*_*ℓ*_1_-MTL method, which concatenated the multiple modalities features into a long vector features.

The prediction performance results are shown in Tables 3 and 4. From the results, it is clear that the methods with multimodality outperform the methods using one single modality of data. This validates our assumption that the complementary information among different modalities is helpful for cognitive function prediction. Regardless of two or three modalities, *ℓ*_2,1_*ℓ*_*q*_-MKMTL achieved better performances than the linear based multitask learning for the most cases, the same as for the single modality learning task above.

## 6. Conclusion

Many multitask learning methods with sparsity-inducing regularization for modeling AD cognitive outcomes have been proposed in the past decades. However, the current formulations remain restricted to the linear models and cannot capture the relationship between the MRI features and cognitive outcomes. To address these shortcomings, we applied two multikernel multitask learning methods with a joint sparsity-inducing regularization to model the more complicated but more flexible relationship between MRI features and cognitive outcomes and demonstrated their effectiveness compared with linearized multitask learning methods by applying them to the ADNI data for predicting cognitive outcomes from MRI scans. Extensive experiments on ADNI dataset illustrate that the multikernel multitask learning method not only yields superior performance on regression performance but also is a powerful tool for fusing multimodalities data.

## Figures and Tables

**Algorithm 1:**
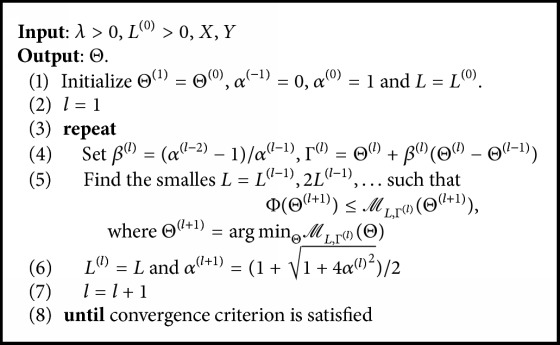
*ℓ*_*q*_ *ℓ*_1_-MTL.

**Algorithm 2:**
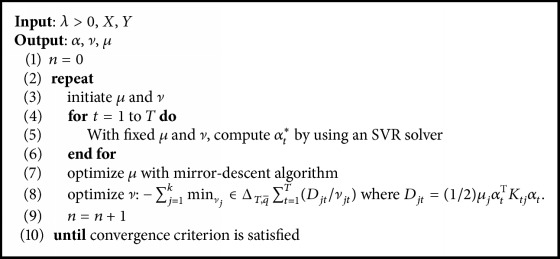
*ℓ*_*q*_ *ℓ*_1_-MKMTL.

**Algorithm 3:**
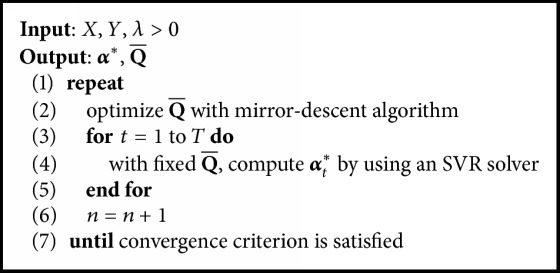
*ℓ*_2,1_-*ℓ*_*q*_-MKMTL.

**Table 1:** Performance comparison of various methods in terms of rMSE and nMSE on 10 cross validation cognitive prediction tasks.

Method	ADAS	MMSE	RAVLT			
			TOTAL	TOT6	T30	RECOG
Ridge	7.556 ± 0.294	2.656 ± 0.134	11.41 ± 0.498	3.907 ± 0.236	4.052 ± 0.224	4.331 ± 0.294
Lasso	6.846 ± 0.361	2.216 ± 0.098	10.02 ± 0.548	3.320 ± 0.195	3.443 ± 0.177	3.639 ± 0.213
MKL	6.893 ± 0.528	2.214 ± 0.106	9.911 ± 0.695	3.424 ± 0.296	3.570 ± 0.340	3.745 ± 0.237
Robust MTL	7.651 ± 0.442	3.326 ± 0.266	11.02 ± 0.590	3.574 ± 0.235	3.704 ± 0.171	3.858 ± 0.310
CMTL	7.642 ± 0.373	3.083 ± 0.461	11.56 ± 0.510	3.907 ± 0.260	4.038 ± 0.244	4.381 ± 0.226
Trace	8.180 ± 0.605	6.113 ± 2.038	13.09 ± 3.128	3.782 ± 0.491	3.906 ± 0.431	4.520 ± 0.859
SRMTL	6.882 ± 0.325	2.331 ± 0.271	9.961 ± 0.561	3.320 ± 0.152	3.445 ± 0.116	3.639 ± 0.261
*ℓ*_*q*_ *ℓ*_1_-MTL	6.772 ± 0.312	2.206 ± 0.081	9.606 ± 0.448	3.344 ± 0.154	3.440 ± 0.151	3.644 ± 0.247
*ℓ*_*q*_ *ℓ*_1_-MKMTL	6.825 ± 0.455	2.417 ± 0.197	9.699 ± 0.505	3.396 ± 0.188	3.495 ± 0.144	3.653 ± 0.243
*ℓ*_2,1_ *ℓ*_*q*_-MKMTL	6.806 ± 0.447	2.185 ± 0.106	9.628 ± 0.510	3.331 ± 0.196	3.467 ± 0.172	3.627 ± 0.199

Method	FLU		TRAILS		nMSE	
	ANIM	VEG	A	B		

Ridge	6.521 ± 0.418	4.322 ± 0.178	27.18 ± 1.702	83.72 ± 5.713	16.44 ± 1.725	
Lasso	5.352 ± 0.447	3.701 ± 0.093	23.75 ± 1.398	71.23 ± 2.812	12.05 ± 0.758	
MKL	5.342 ± 0.510	3.761 ± 0.137	24.71 ± 1.781	78.09 ± 6.916	13.56 ± 1.133	
Robust MTL	5.946 ± 0.398	3.988 ± 0.083	27.78 ± 1.922	90.12 ± 7.098	17.68 ± 2.303	
CMTL	6.608 ± 0.561	4.398 ± 0.284	27.46 ± 1.980	83.66 ± 5.418	16.67 ± 1.912	
Trace	6.743 ± 1.425	4.672 ± 0.778	28.82 ± 3.278	89.68 ± 7.838	20.23 ± 5.215	
SRMTL	5.327 ± 0.334	3.713 ± 0.088	25.09 ± 1.421	80.00 ± 4.637	14.01 ± 1.169	
*ℓ*_*q*_ *ℓ*_1_-MTL	5.298 ± 0.439	3.704 ± 0.096	23.42 ± 1.110	71.32 ± 2.945	11.92 ± 0.969	
*ℓ*_*q*_ *ℓ*_1_-MKMTL	5.304 ± 0.350	3.676 ± 0.094	23.09 ± 1.438	70.28 ± 0.898	11.72 ± 0.222	
*ℓ*_2,1_ *ℓ*_*q*_-MKMTL	5.232 ± 0.434	3.675 ± 0.157	23.13 ± 1.473	69.82 ± 1.236	**11.56** ± **0.602**	

**Table 2:** Performance comparison of various methods in terms of CC and wR on 10 cross validation cognitive prediction tasks.

Method	ADAS	MMSE	RAVLT			
			TOTAL	TOT6	T30	RECOG
Ridge	0.603 ± 0.031	0.407 ± 0.040	0.401 ± 0.084	0.361 ± 0.092	0.377 ± 0.096	0.261 ± 0.080
Lasso	0.655 ± 0.036	0.540 ± 0.046	0.493 ± 0.084	0.507 ± 0.100	0.523 ± 0.106	0.416 ± 0.087
MKL	0.658 ± 0.030	0.544 ± 0.052	0.502 ± 0.066	0.476 ± 0.095	0.506 ± 0.105	0.391 ± 0.072
Robust MTL	0.587 ± 0.022	0.338 ± 0.084	0.423 ± 0.090	0.432 ± 0.096	0.444 ± 0.094	0.354 ± 0.105
CMTL	0.603 ± 0.025	0.381 ± 0.042	0.397 ± 0.072	0.362 ± 0.090	0.381 ± 0.099	0.260 ± 0.068
Trace	0.548 ± 0.039	0.144 ± 0.091	0.342 ± 0.172	0.395 ± 0.159	0.402 ± 0.142	0.253 ± 0.130
SRMTL	0.655 ± 0.034	0.525 ± 0.058	0.492 ± 0.079	0.505 ± 0.097	0.523 ± 0.103	0.413 ± 0.092
*ℓ*_*q*_ *ℓ*_1_-MTL	0.662 ± 0.043	0.532 ± 0.056	0.532 ± 0.082	0.492 ± 0.109	0.522 ± 0.105	0.404 ± 0.091
*ℓ*_*q*_ *ℓ*_1_-MKMTL	0.661 ± 0.034	0.460 ± 0.099	0.519 ± 0.072	0.470 ± 0.089	0.494 ± 0.094	0.412 ± 0.090
*ℓ*_2,1_ *ℓ*_*q*_-MKMTL	0.660 ± 0.035	0.547 ± 0.045	0.529 ± 0.079	0.500 ± 0.095	0.508 ± 0.094	0.421 ± 0.075

Method	FLU		TRAILS		wR	
	ANIM	VEG	A	B		

Ridge	0.185 ± 0.090	0.396 ± 0.073	0.291 ± 0.097	0.330 ± 0.110	0.361 ± 0.041	
Lasso	0.365 ± 0.096	0.506 ± 0.059	0.363 ± 0.041	0.467 ± 0.096	0.484 ± 0.049	
MKL	0.375 ± 0.071	0.496 ± 0.067	0.374 ± 0.056	0.457 ± 0.060	0.478 ± 0.046	
Robust MTL	0.253 ± 0.096	0.443 ± 0.057	0.282 ± 0.113	0.292 ± 0.123	0.385 ± 0.038	
CMTL	0.180 ± 0.089	0.390 ± 0.071	0.287 ± 0.116	0.335 ± 0.112	0.358 ± 0.036	
Trace	0.212 ± 0.143	0.331 ± 0.112	0.270 ± 0.112	0.290 ± 0.122	0.319 ± 0.083	
SRMTL	0.362 ± 0.093	0.503 ± 0.064	0.340 ± 0.063	0.361 ± 0.095	0.468 ± 0.045	
*ℓ*_*q*_ *ℓ*_1_-MTL	0.379 ± 0.076	0.501 ± 0.063	0.399 ± 0.060	0.467 ± 0.098	0.489 ± 0.050	
*ℓ*_*q*_ *ℓ*_1_-MKMTL	0.381 ± 0.080	0.521 ± 0.067	0.421 ± 0.064	0.481 ± 0.076	0.482 ± 0.047	
*ℓ*_2,1_ *ℓ*_*q*_-MKMTL	0.409 ± 0.073	0.516 ± 0.065	0.417 ± 0.067	0.490 ± 0.087	**0.500** ± **0.043**	

**Table 3:** Performance comparison of various methods with fusing multiple modalities data in terms of rMSE and nMSE on 10 cross validation cognitive prediction tasks.

Method	ADAS	MMSE	FLU	TRAILS	
			ANIM	A	B
*ℓ*_*q*_ *ℓ*_1_-MTL-MRI	6.494 ± 1.029	1.964 ± 0.306	4.911 ± 0.256	16.39 ± 2.906	55.82 ± 7.689
*ℓ*_*q*_ *ℓ*_1_-MTL-PET	6.941 ± 1.244	2.118 ± 0.298	5.192 ± 0.145	16.56 ± 3.533	56.88 ± 9.447
*ℓ*_*q*_ *ℓ*_1_-MTL-MP	6.219 ± 1.037	2.067 ± 0.293	4.928 ± 0.260	16.09 ± 2.768	53.70 ± 7.144
*ℓ*_*q*_ *ℓ*_1_-MTL-ALL	6.174 ± 0.978	2.062 ± 0.272	4.789 ± 0.206	15.97 ± 2.785	53.37 ± 7.243
*ℓ*_*q*_ *ℓ*_1_-MKMTL-MRI	6.369 ± 0.941	2.074 ± 0.291	4.993 ± 0.235	16.18 ± 3.089	55.95 ± 9.479
*ℓ*_*q*_ *ℓ*_1_-MKMTL-PET	6.812 ± 1.155	2.060 ± 0.364	5.151 ± 0.227	16.61 ± 3.588	57.85 ± 11.24
*ℓ*_*q*_ *ℓ*_1_-MKMTL-MP	6.112 ± 0.886	2.005 ± 0.258	4.966 ± 0.269	16.13 ± 2.988	54.13 ± 9.450
*ℓ*_*q*_ *ℓ*_1_-MKMTL-ALL	5.960 ± 0.834	1.959 ± 0.256	4.821 ± 0.224	16.00 ± 3.062	53.48 ± 9.592
*ℓ*_2,1_ *ℓ*_*q*_-MKMTL-MRI	6.425 ± 0.951	1.951 ± 0.308	4.886 ± 0.264	16.11 ± 2.939	54.96 ± 7.499
*ℓ*_2,1_ *ℓ*_*q*_-MKMTL-PET	6.783 ± 1.059	2.058 ± 0.323	5.107 ± 0.258	16.52 ± 3.515	55.51 ± 9.568
*ℓ*_2,1_ *ℓ*_*q*_-MKMTL-MP	6.086 ± 0.987	1.917 ± 0.299	4.855 ± 0.249	15.95 ± 2.996	52.44 ± 8.074
*ℓ*_2,1_ *ℓ*_*q*_-MKMTL-ALL	6.034 ± 0.978	1.905 ± 0.294	4.809 ± 0.244	15.88 ± 3.028	52.20 ± 8.120

Method	RAVLT				nMSE
	TOTAL	TOT6	T30	RECOG	

*ℓ*_*q*_ *ℓ*_1_-MTL-MRI	10.18 ± 0.640	3.538 ± 0.147	3.735 ± 0.199	3.169 ± 0.306	10.24 ± 0.735
*ℓ*_*q*_ *ℓ*_1_-MTL-PET	10.41 ± 0.441	3.627 ± 0.140	3.796 ± 0.176	3.258 ± 0.360	10.72 ± 1.163
*ℓ*_*q*_ *ℓ*_1_-MTL-MP	10.01 ± 0.556	3.501 ± 0.149	3.693 ± 0.196	3.164 ± 0.314	9.710 ± 0.627
*ℓ*_*q*_ *ℓ*_1_-MTL-ALL	9.755 ± 0.575	3.450 ± 0.151	3.643 ± 0.200	3.172 ± 0.313	9.525 ± 0.608
*ℓ*_*q*_ *ℓ*_1_-MKMTL-MRI	10.09 ± 0.605	3.532 ± 0.081	3.731 ± 0.253	3.203 ± 0.304	10.21 ± 1.019
*ℓ*_*q*_ *ℓ*_1_-MKMTL-PET	10.30 ± 0.436	3.592 ± 0.145	3.754 ± 0.231	3.200 ± 0.357	10.82 ± 1.455
*ℓ*_*q*_ *ℓ*_1_-MKMTL-MP	9.787 ± 0.375	3.471 ± 0.089	3.664 ± 0.199	3.159 ± 0.302	9.713 ± 0.968
*ℓ*_*q*_ *ℓ*_1_-MKMTL-ALL	9.350 ± 0.460	3.402 ± 0.030	3.604 ± 0.221	3.196 ± 0.291	9.410 ± 0.985
*ℓ*_2,1_ *ℓ*_*q*_-MKMTL-MRI	9.984 ± 0.525	3.477 ± 0.130	3.678 ± 0.204	3.143 ± 0.314	9.937 ± 0.753
*ℓ*_2,1_ *ℓ*_*q*_-MKMTL-PET	10.19 ± 0.410	3.565 ± 0.146	3.745 ± 0.212	3.191 ± 0.351	10.31 ± 1.105
*ℓ*_2,1_ *ℓ*_*q*_-MKMTL-MP	9.727 ± 0.467	3.397 ± 0.136	3.593 ± 0.162	3.112 ± 0.323	9.282 ± 0.869
*ℓ*_2,1_ *ℓ*_*q*_-MKMTL-ALL	9.561 ± 0.442	3.361 ± 0.124	3.556 ± 0.170	3.104 ± 0.327	**9.160** ± **0.860**

**Table 4:** Performance comparison of various methods with fusing multiple modalities data in terms of CC and wR on 10 cross validation cognitive prediction tasks.

Method	ADAS	MMSE	FLU	TRAILS	
			ANIM	A	B
*ℓ*_*q*_ *ℓ*_1_-MTL-MRI	0.670 ± 0.091	0.539 ± 0.117	0.481 ± 0.112	0.417 ± 0.115	0.525 ± 0.073
*ℓ*_*q*_ *ℓ*_1_-MTL-PET	0.619 ± 0.058	0.482 ± 0.087	0.395 ± 0.105	0.385 ± 0.120	0.501 ± 0.060
*ℓ*_*q*_ *ℓ*_1_-MTL-MP	0.700 ± 0.070	0.549 ± 0.108	0.486 ± 0.119	0.437 ± 0.119	0.567 ± 0.070
*ℓ*_*q*_ *ℓ*_1_-MTL-ALL	0.705 ± 0.067	0.560 ± 0.096	0.527 ± 0.102	0.450 ± 0.115	0.575 ± 0.064
*ℓ*_*q*_ *ℓ*_1_-MKMTL-MRI	0.677 ± 0.093	0.512 ± 0.113	0.464 ± 0.095	0.411 ± 0.113	0.529 ± 0.094
*ℓ*_*q*_ *ℓ*_1_-MKMTL-PET	0.634 ± 0.056	0.493 ± 0.100	0.410 ± 0.133	0.375 ± 0.090	0.478 ± 0.061
*ℓ*_*q*_ *ℓ*_1_-MKMTL-MP	0.710 ± 0.060	0.537 ± 0.106	0.472 ± 0.111	0.426 ± 0.105	0.566 ± 0.081
*ℓ*_*q*_ *ℓ*_1_-MKMTL-ALL	0.727 ± 0.062	0.551 ± 0.112	0.512 ± 0.097	0.444 ± 0.099	0.582 ± 0.065
*ℓ*_2,1_ *ℓ*_*q*_-MKMTL-MRI	0.673 ± 0.096	0.548 ± 0.124	0.491 ± 0.095	0.422 ± 0.135	0.528 ± 0.102
*ℓ*_2,1_ *ℓ*_*q*_-MKMTL-PET	0.631 ± 0.057	0.488 ± 0.108	0.418 ± 0.119	0.386 ± 0.095	0.524 ± 0.065
*ℓ*_2,1_ *ℓ*_*q*_-MKMTL-MP	0.714 ± 0.067	0.566 ± 0.107	0.499 ± 0.094	0.437 ± 0.122	0.583 ± 0.077
*ℓ*_2,1_ *ℓ*_*q*_-MKMTL-ALL	0.721 ± 0.064	0.574 ± 0.105	0.512 ± 0.094	0.445 ± 0.120	0.589 ± 0.073

Method	RAVLT				wR
	TOTAL	TOT6	T30	RECOG	

*ℓ*_*q*_ *ℓ*_1_-MTL-MRI	0.576 ± 0.077	0.536 ± 0.085	0.516 ± 0.041	0.444 ± 0.079	0.523 ± 0.082
*ℓ*_*q*_ *ℓ*_1_-MTL-PET	0.548 ± 0.103	0.497 ± 0.124	0.490 ± 0.092	0.409 ± 0.098	0.481 ± 0.081
*ℓ*_*q*_ *ℓ*_1_-MTL-MP	0.593 ± 0.079	0.547 ± 0.086	0.529 ± 0.038	0.450 ± 0.075	0.540 ± 0.077
*ℓ*_*q*_ *ℓ*_1_-MTL-ALL	0.618 ± 0.072	0.563 ± 0.077	0.546 ± 0.027	0.446 ± 0.085	0.554 ± 0.069
*ℓ*_*q*_ *ℓ*_1_-MKMTL-MRI	0.585 ± 0.069	0.533 ± 0.093	0.511 ± 0.044	0.434 ± 0.077	0.517 ± 0.079
*ℓ*_*q*_ *ℓ*_1_-MKMTL-PET	0.559 ± 0.110	0.508 ± 0.111	0.503 ± 0.085	0.432 ± 0.081	0.488 ± 0.075
*ℓ*_*q*_ *ℓ*_1_-MKMTL-MP	0.617 ± 0.080	0.561 ± 0.100	0.541 ± 0.057	0.462 ± 0.079	0.543 ± 0.075
*ℓ*_*q*_ *ℓ*_1_-MKMTL-ALL	0.654 ± 0.071	0.577 ± 0.082	0.560 ± 0.038	0.444 ± 0.087	0.561 ± 0.068
*ℓ*_2,1_ *ℓ*_*q*_-MKMTL-MRI	0.594 ± 0.070	0.554 ± 0.080	0.536 ± 0.033	0.459 ± 0.071	0.534 ± 0.082
*ℓ*_2,1_ *ℓ*_*q*_-MKMTL-PET	0.563 ± 0.104	0.510 ± 0.111	0.501 ± 0.081	0.436 ± 0.095	0.495 ± 0.072
*ℓ*_2,1_ *ℓ*_*q*_-MKMTL-MP	0.621 ± 0.075	0.582 ± 0.083	0.564 ± 0.046	0.475 ± 0.073	0.560 ± 0.071
*ℓ*_2,1_ *ℓ*_*q*_-MKMTL-ALL	0.637 ± 0.068	0.593 ± 0.077	0.575 ± 0.041	0.479 ± 0.081	**0.570** ± **0.067**
